# Anogenital Measurements in Newborns

**DOI:** 10.4274/jcrpe.604

**Published:** 2012-03-08

**Authors:** Behzat Özkan

**Affiliations:** 1 İstanbul Medeniyet University Faculty of Medicine, Department of Pediatric Endocrinology, İstanbul, Turkey; ozkan.behzat@gmail.com

**Keywords:** Newborns, caliper, Anogenital distance

## INTRODUCTION

We would like to thank Oguz Kutlu A for her close interest in our paper entitled “Anogenital Distance in Turkish Newborns”. In our study (1), we used caliper for the measurements as Oguz Kutlu A did in her study (2). We compared our anogenital measurements to those obtained in previous studies in the literature (including Oguz Kutlu’s study) according to the measurement method used, as shown in the table below. Although different measurement methods were applied, our results were similar to those from the mentioned studies. Variations in the results reported can be due to genetic and ethnic factors, as well as to the measurement methods used and the age of the newborn at the time of the measurements, as it was emphasized in the discussion section of our paper (3). However, anogenital distance measurement method used in the study by Oguz Kutlu A, was wrongly written as tape measurement instead of caliper measurement (2). We are, again, giving the original table including correct measurement technique belonging to Oguz Kutlu A. In addition, I would like to thank her for giving extra information about the genital measurements in the Letter. 

## Figures and Tables

**Table 1:**
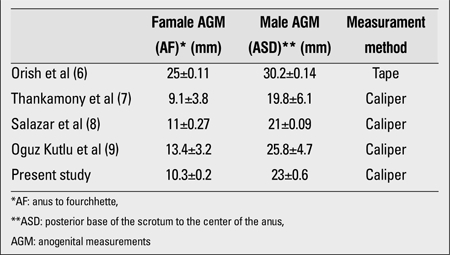
Comparison of AGM measurements according to themeasurement method used (mean±SD)
